# Human Gender Differences in the Perception of Conspecific Alarm Chemosensory Cues

**DOI:** 10.1371/journal.pone.0068485

**Published:** 2013-07-24

**Authors:** Anca R. Radulescu, Lilianne R. Mujica-Parodi

**Affiliations:** 1 Department of Mathematics, University of Colorado, Boulder, Colorado, United States of America; 2 Department of Biomedical Engineering, Stony Brook University, Stony Brook, New York, United States of America; CNRS, France

## Abstract

It has previously been established that, in threatening situations, animals use alarm pheromones to communicate danger. There is emerging evidence of analogous chemosensory “stress” cues in humans. For this study, we collected alarm and exercise sweat from “donors,” extracted it, pooled it and presented it to 16 unrelated “detector” subjects undergoing fMRI. The fMRI protocol consisted of four stimulus runs, with each combination of stimulus condition and donor gender represented four times. Because olfactory stimuli do not follow the canonical hemodynamic response, we used a model-free approach. We performed minimal preprocessing and worked directly with block-average time series and step-function estimates. We found that, while male stress sweat produced a comparably strong emotional response in both detector genders, female stress sweat produced a markedly stronger arousal in female than in male detectors. Our statistical tests pinpointed this gender-specificity to the right amygdala (strongest in the superficial nuclei). When comparing the olfactory bulb responses to the corresponding stimuli, we found no significant differences between male and female detectors. These imaging results complement existing behavioral evidence, by identifying whether gender differences in response to alarm chemosignals are initiated at the perceptual versus emotional level. Since we found no significant differences in the olfactory bulb (primary processing site for chemosensory signals in mammals), we infer that the specificity in responding to female fear is likely based on processing meaning, rather than strength, of chemosensory cues from each gender.

## Introduction

It has been established for a few decades that animals (from insects to mammals) are able to communicate through body “odor,” not only reproductive state, but also socially relevant emotions like alarm [1], fear [2], aggression [3], anxiety [4]. These chemosensory signals (pheromones) induce corresponding physiological and behavioral adaptations within surrounding conspecifics. They are conveyed, in addition to the main olfactory system, by known specialized sensory systems (e.g., vomeronasal organ, trance-amine-associated receptors, Gruenberg ganglion cells). While work starting in 1981, with a pioneering study by Owen [5], has been testing the existence of these mechanisms within human physiology [6], [7], the extension to humans of pheromone-driven behavior and specialized pathways remained for a long time subject to controversy [8].

Over the past two decades, more reliable scientific evidence has been gathered, supporting the theory that each human has a unique chemosensory signature that carries information related to their genetic, personal and environmental variables [9]. In humans, the neuronal underpinnings of the processing of social chemosignals have been investigated in relation to kin recognition, mate choice, the reproductive state and emotional contagion [10]. Human body odors were shown to carry information about potential partners in terms of gender and sexual orientation [11].

In particular, the ability to communicate anxiety through chemosensory signals has been documented in humans by behavioral, perceptual and brain imaging studies [12]. It has been demonstrated that the human brain responds to conspecific fear signals encoded within chemosensory cues, which are processed differently than other perceptually similar odors [9]. De Groot et al underlined the social relevance of chemosignals in regulating communicative correspondence outside of conscious access [13], by showing that fear chemosignals generate a fearful facial expression and sensory acquisition, while disgust chemosignals evoke a disgusted facial expression and sensory rejection. The reports on human chemosensory communication of stress signaling have typically used sweat as the chemosensory stimulus [14], collected via axillary pads, with the donors' emotional state being induced, for example, by watching a distressful movie [15], [2], or by preparing a difficult examination [16].

To verify whether these stimuli elicited an emotional response in the detectors, olfactory stimulation has often been coupled with means of emotional and cognitive evaluation, such as word-association [17], or presentation of images (e.g., pictures of facial affect) with an emotional priming task [16]. Smelling sweat from donors who had been watching an emotionally stimulating movie was found to improve accuracy in completing a word-association task [17]. In conjunction with emotional priming, stress sweat was found to cause participants to interpret ambiguous expressions as more fearful [18]. Females exposed to the stress odor were less likely to judge a face as positive when primed with a positive face [16]. When communicated between males, anxiety chemosignals were shown to influence the perception of emotional (happy) faces [19].

While such a variety of studies have studied the behavioral effects of such chemosensory cues, only a few have investigated their central processing. These studies reveal that human chemosignals are probably processed not only within olfactory brain areas, but also through neuronal relays responsible for the processing of social information [10]. The chemosensory perception of human anxiety seems to automatically recruit empathy-related resources. Indeed, an fMRI study by Prehn-Kristensen et al [20] shows that chemosensory anxiety signals activate brain areas involved in the processing of social emotional stimuli (fusiform gyrus), and in the regulation of empathic feelings (insula, precuneus, cingulate cortex). In addition, neuronal activity within attentional (thalamus, dorsomedial prefrontal cortex) and emotional (cerebellum, vermis) control systems were observed. An interesting fact is that anxiety and anxiety-related chemosignals as well as decision-making share similar regions of neuronal activation. A study by Haegler et al [21] evidenced that chemosensory anxiety signals communicated between humans can increasing participants' risk-taking behavior.

Along these lines, a study by Mujica-Parodi et al [22] investigated how fear sweat activates limbic areas of the brain, regions associated with emotional processing. For this study, sweat samples were collected from donors (male and female), while undergoing an acute emotional stressor (first time skydive), were pooled according to gender and were presented, in odorless form, blind to condition and with exercise sweat as control, to a separate group of subjects (male and female). Cortisol levels and self-reported state anxiety confirmed that the experimental paradigm induced in the donor subjects an intense state of emotional stress and that exercise provided an acceptable control [22]. The authors then investigated the reaction of the amygdala in response to smelling the sweat “stress cues.” The amygdala was chosen as the region of interest based on its well-known association with emotion in general, on its key role in olfactory processing [23] and on its implication in the emotional processing of other (alarm, sexual) olfactory stimuli (newer studies [24] have found different subdivisions to be specialized in processing respectively odors and odorless stimuli). It has been suggested [25] that the amygdala codes neither intensity nor valence per se, but a combination that was suggested to reflect the overall emotional value of a stimulus. The analysis of fMRI time series demonstrated amygdala activation in detectors in response to unconscious inhalation of alarm sweat, thus strongly supporting existence of human chemosignals communicating alarm, and their ability to inherently elicit an emotional response.

While hypotheses on human chemosignals have been recently gaining ground, results on gender differences in response to chemosensori stimuli from conspecifics (alarm pheromones in particular, human or animal) have been initially overlooked and even disavowed. In a 2000 study on alarm pheromones in salamanders, “subjects were not sexed, because there was no reason to believe that response should be affected by the sex of emitters or receivers” [26]); however, a 2006 study on crucian carp found remarkable differences between males and females in the discriminatory power of the olfactory neurons toward sex pheromones [27]. Other studies have also suggested gender among the factors that may influence reception and behavior in response to human putative pheromones [28]. In line with research on general olfactory perception (which demonstrated better perception in females than in males [29]), a study by Chen and Haviland-Jones [15] concluded that females performed better than males in identifying the emotional state of donors from their body odor. Pause et al., in a study including both gender subjects, were able to demonstrate only in females the effects of inhaling alarm pheromones on emotional priming of faces [16]. A later study analyzing chemosensory event-related potentials (in response to sweat stimuli collected from humans awaiting a stressful examination) found that neuronal brain activity in males was much weaker than in females [12]. Male chemosensory anxiety signals compared with neutral chemosignals triggered increased state anxiety in female receivers, in a recent study by Albrecht et al [30]. However, what remains open to investigation is the question of whether the gender discrepancies were elicited by differences in sensitivity to negative emotional stimulation, or to the chemosignals *per se*.

**Figure 1:**
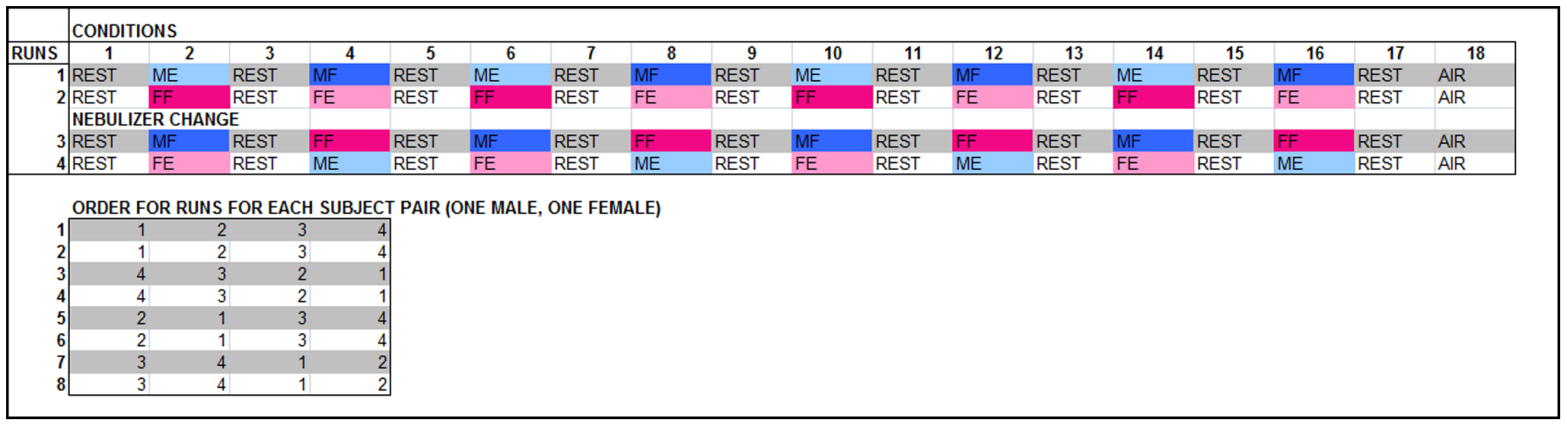
Study design. We presented each fMRI subject (detector) with 16 stimulus blocks, so that each possible combination of donor gender and condition (ME = male exercise, MF = male fear, FE = female exercise, FF = female fear) was represented four times. Air was presented at the beginning and the end (A = Air), and between each two stimulus blocks (R = Rest). The gender of the detectors alternates (odd-index subjects are male, and even-index subjects are female. Different permutations of the runs were used for each fMRI subject per gender group, to eliminate possible order confounds.

In the current work, we further address precisely these questions. We performed a reinvestigation of a data-set which has previously been presented [22], this time using a less conventional methodology, aimed to better make use of a design specifically created to optimize between-gender comparisons. While in the fMRI scanner, the 16 detectors (8 male and 8 female) breathed continuously through an olfactometer, while presented with the chemosensory stimuli: fear (F) and exercise (E) stimuli, extracted from the sweat of 40 male (M) and female (F) donors, while skydiving (fear) and respectively exercising on a treadmill (exercise). Over a total of four runs, the detectors received a train of 16 stimulus blocks, encompassing four times each possible combination of donor gender and condition (ME = male exercise, MF = male fear, FE = female exercise, FF = female fear), with air presented at the beginning and the end (A = Air), and between each two stimulus blocks (R = Rest) (see Figure 1; also see the Methods section and [22] for further details).

**Figure 2:**
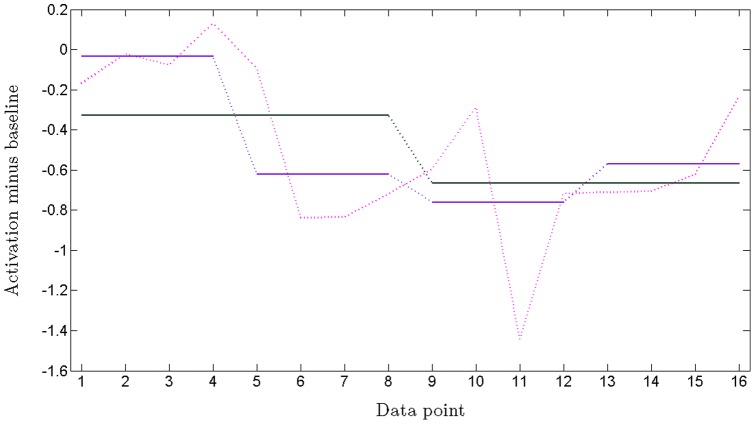
Step function approximations for the 16 data-points time series that represents the response to a stimulus block. The original time series (pink) represents the average right amygdala response in one subject, when exposed to MF stimuli, and includes the duration on the stimulus block (first 8 data-points) and the following rest block (next 8 data-points). We show examples of approximations of this curve by step functions, averaging over 4 half-block segments (purple) and averaging over the entire blocks (black). These approximations, as well as the mean value of the series, are more stable to variability than the original time-series, and were therefore used as “surrogates” of the time series when performing the between-group statistical comparisons.

The original analysis [22] focused on the more general identification of an amygdala response in reaction to alarm chemosignals, irrespective of gender. Here, we complement this first study by investigating (1) whether the amygdala response exhibited any gender differences (with respect to both donors and detectors), and (2) whether these differences originated at the perceptual or emotional level. For each detector, we considered the four average, block-specific time series (ME, MF, FE and FF). We studied these time series over four regions of interest (ROIs): the bilateral amygdala with its subdivisions, and the bilateral olfactory bulb, the primary olfactory projection area (see Figure 2 for additional detail on the time series). The amygdala was chosen based on our previous findings and in light of its involvement in emotional processing of odors [31]; the olfactory bulb was additionally observed in order to verify whether any differences in amygdala activation (i.e., the emotional aspects of the circuitry) were potentially subsequent to discrepancies at the level of stimulus perception.

## Results

### Differences in amygdala activation based on detector gender

**Figure 3:**
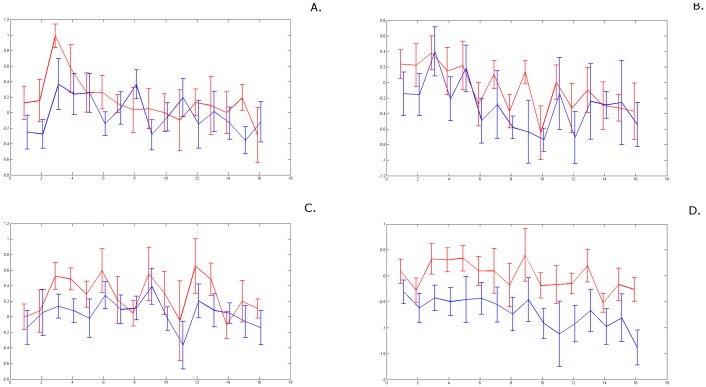
Response to alarm chemosignals: to MF (top) and FF (bottom), further separated by the gender of the detector. *Females*' *responses are shown in red, and males*' *responses are shown in blue.* **Left.** *Left amygdala.* **Right.** *Right amygdala.*

We performed a direct comparison between amygdala responses to FF and MF, separated by detector gender (Figure 3). For our analysis, we considered two-block time series: the activation block (8 data-points, 20 seconds) followed by the corresponding rest block. We found that the reaction to MF was comparable in male and female detectors, both for the left and the right amygdala. However, when we compared the males' and females' response to FF, we found the difference to be significant for the right amygdala (see Figure 3d) with the average for male detectors (blue) clearly lower than the average for female detectors (red). To test this result statistically, we performed a triple Wilcoxon rank comparison between the two FF block average time series, as described in Table 1, and further explained in the Methods section: (1) we compared means taken separately over four half-blocks (10 sec intervals); (2) we compared means taken over each of the two blocks (20 sec intervals); (3) we compared the mean values for the entire two-block time series. As shown by Figure 1, the statistics support gender differences, with the FF stimulus (female fear/alarm) eliciting a significantly lower right amygdala response in males than in females.

**Table 1:** Gender differences in detectors' right amygdala activation, in response to both Male and Female Fear.

	Male Fear								Rest								Female Fear								Rest							
	1	2	3	4	5	6	7	8	1	2	3	4	5	6	7	8	1	2	3	4	5	6	7	8	1	2	3	4	5	6	7	8
LA	0.23				0.13				0.03				0.02^*^				0.19				0.64				0.44				0.38			
	0.27								0.72								0.27								0.82							
	0.50																0.64															
RA	0.16				0.08^*^				0.02^*^				0.32				0.01^**^				0.04^*^				0.007^**^				0.007^**^			
	0.13								0.95								0.04^*^								0.04^*^							
	0.57																0.04^*^															

We show statistical differences (p-values) between amygdala activation for male and female detectors in response to Male Fear (left side) and Female Fear (right side). Each column of the table represents a data-point (the first 8 data-points, denoted by Male Fear 1–8, respectively by Female Fear 1–8, represent the stimulus block; the next 8 data-points, denoted by R1-R8, represent the following Rest block). The top four rows summarize the differences in the left amygdala (LA), the bottom four rows describe the differences found in the right amygdala. For each case, the first row shows the p-values for averages take over 4 data-point half-blocks (4 segments ⇒ 4 p-values); the second row – the p-values for averages take over entire 8 data-point blocks (2 blocks ⇒ 2 p-values); the tird row – the p-value for average of the entire 16 data-point time series.

### Subregion-specific differences in right amygdala

**Figure 4:**
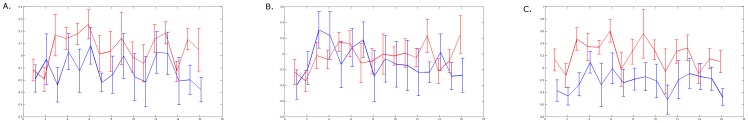
Gender differences in detectors in response to Female Fear, broken down for right amygdala subdivisions. *Females*' *responses are shown in red, and males*' *responses are shown in blue.* **Left**: *latero-basal (RA-LB);* Center: *centro-medial (RA-CM);* **Right**: *superficial (RA-SF).*

We further investigated whether this reaction was triggered specifically by any right amygdala subdivision. We performed a similar statistical analysis, breaking the amygdala down into three subdivisions (latero-basal, centro-medial and superficial), known to have finely distinct contributions to the processing of both olfactory stimuli and alarm. We found that the effect was strongly driven by the superficial nuclei of the right amygdala, and to a lesser extent (trend) by the latero-basal amygdala; in the centro-medial subdivision there were no differences. This is illustrated in Figure 4, and the statistical results are presented in Table 2.

**Table 2:** Gender differences in detectors in response to Female Fear, by right amygdala subdivisions.

		1	2					3				4			5					6								7							8			1							2	3									4			5			6								7				8	
RA-LB		0.72	1					0.06				0.79				0.32				0.50							0.13								0.44				0.95						0.95	0.95								0.50				0.27					0.44						0.02				0.23	
		0.23														0.72																							0.79																			0.13																
		0.19																																					0.44																																			
		0.23																																																																								
RA-CM		0.50		0.95				0.44				0.57			0.38					0.64								0.32							0.72			0.95							0.70	0.32									0.38			0.03			0.44								0.32				0.16	
		0.38													0.72																							0.72																				0.95																
	0.87																																				0.32																																					
	0.79																																																																									
RA-SF	0.13		0.23		0.02						0.32		0.03					0.13					0.50										0.10				0.27				0.10									0.38		0.27				0.44			0.72								0.16					0.01		
	0.01												0.04																								0.04																			0.02																		
	0.01																																				0.02																																					
	0.007																																																																									

Statistical differences (p-values) between activation in right amygdala subdivisions, for male and female detectors in response to Female Fear (FF) stimuli, further broken down for right amygdala subdivisions: latero-basal (RA–LB), centro-medial (RA–CM) and superficial (RA–SF).

### Results in the olfactory bulb

Comparing olfactory bulb responses to FF and MF, we found no significant differences between the male and female detectors.

## Discussion

Our results support gender differences in response to alarm chemosensory cues. We found that while male alarm produced a comparably strong emotional response in both detector genders, female alarm produced significantly increased arousal in female than in male detectors (male detectors overall ignoring female alarm as a less emotionally charged stimulus). When proceeding to narrowing down the location of this effect within the right amygdala, we found the gender differences to be most pronounced in the superficial nuclei (which is also where [22] had found, performing a standard analysis, the stronger amygdala activity in response to alarm chemosensory cues). In the existing literature on both animals and humans, the exact functional distribution within the amygdala is still under investigation [32]. While some studies propose the centro-medial baso-lateral amygdalae as the centers for olfactory emotional processing [24], [33], our results are in line with other studies supporting the involvement of superficial (cortical) nuclei in the processing of olfactory [34], [35] and other chemosensory signals [36] (alarm chemosensory cues in particular [37], [38]).

A typical treatment of fMRI time series (in particular, our previous work on the data-set [22]) uses the General Linear Model of SPM as an underlying model for the data. Assumptions have to be made on the duration and shape of the hemodynamic response function (e.g., 20 sec HRF, and 4 Bessel base functions, in our case); these improve, by underlying a Baysian model to the data, the statistical power otherwise restricted by the limited resolution, or sample size. In our current analysis, we made no *a priori* assumptions on shape, amplitude or length of the hemodynamic response. Our ROI time series did not necessarily have, for each voxel and each subject, a typical hemodynamic response shape (increasing to a peak, followed by return to baseline). Our prior work [39], [40] suggests, however, that a time series may encode important properties related to the dynamics of the response in that region, even when it does not show “activation” in the conventional sense (traditionally used in neuroimaging).

Our results support existing evidence of gender differences in response to human alarm chemosignals. They should, however, be regarded as a starting point, since they are subject to the limitation of the small sample size used for this study. The sample size's upper limit resulted from a combination between our desire to use a stressor with high face validity, and a low donor/detector ratio (producing olfactory stimuli for 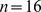 detectors required 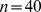 donors). Further work should be able to test larger subject groups, and thus obtain increased statistical power.

Our conclusion is not unexpected in light of existing behavioral studies, describing gender differences in response to stress (*fight-or-flight* versus *tend-and-befriend*). However, while behavioral evidence of the “honey, stop worrying about nothing” effect is important in establishing the existence of gender differences, it does not directly explain where these differences are initiated in the processing sequence of chemosensory stimuli: whether they appear earlier on (as perceptual differences) or later in the sequence (in the emotional processing phases). Indeed, this is a question clearly posed in the existing work of Pause et al., which have found females to respond differently than males to alarm putative pheromones. A study of chemosensory event-related potentials in response to chemosensory anxiety signals found such ERPs to be significantly weaker in males than in females, with socially anxious individuals showing in addition an early processing bias towards signals of social fear, and attentional repression in later processing of the stimulus [12]. In a different study [16], females exposed to the stress odor were less likely to judge a face as positive when primed with a positive face. Pause et al. proposed as potentially contributing causes either that (1) females have a more acute perception than males of putative alarm pheromones from the opposite sex, or (2) the circuitry processing alarm signals (including amygdala) incorporates differently in females the concept of opposite-sex alarm. Both these effects could lead to attending differently to the stimuli. In our current analysis of neuroimaging data, we considered both these possibilities, in order to understand which should be held responsible for the results we had found. Since we found no significant gender differences in the olfactory bulb, which is the primary processing site for both odors and pheromones in mammals [24], we inferred that there is no gender difference in the perception of the stimuli. The gender-specificity in responding to female fear is therefore likely to be based on a difference of emotional processing between genders.

Our findings are in also line with existing rodent work, which ties alarm pheromone production and potency to testosterone [41], thus relating alarm chemosignals to social dominance [42], [43]. Generally, dominant males have more testosterone, hence the alarm signals from dominant males vs. submissive males (or from males vs. females) are likely to be more meaningful (since presumably their threshold to activation is higher). The fact that female chemosignals may be “weaker” meaning-wise is consistent with females showing more consistent detection of such signals. Indeed, while it's well known behaviorally that females in many species have a better “sense of smell” than males, this increased sensitivity may be not perceptual, but rather based on biological meaning, which is not as relevant for males [44].

## Methods – Functional Imaging during Inhalation of Alarm Sweat

### Data acquisition and preprocessing

#### Subjects

This study was approved by the institutional review board at Stony Brook University; all subjects provided written informed consent. Chemosensory alarm/fear and exercise stimuli were extracted from the sweat of 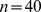 donor subjects of both genders (50% female; see previous published work for details on how the axillary samples were obtained, processed and stored [22]). Detector participants (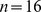) were ages 18–50 (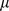 = 26, 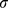 = 3) and were excluded if they had a history of mental illness or substance abuse, neurological illness claustrophobia or metal in the body.

#### Stimuli and testing design

While in the fMRI scanner, the detectors (8 male and 8 female) breathed continuously while presented with the chemosensory stimuli through an olfactometer (no other stimuli were presented during the scans). Four runs were performed, each containing four 20 sec block, representing all combinations of conditions (F, corresponding to presentation of alarm/fear sweat and E, corresponding to presentation of exercise sweat) and donor genders (M = male and F = female), interposed with 20 sec blocks of air. Overall, each combination (ME = male exercise, MF = male fear, FE = female exercise, FF = female fear) was represented four times, with runs counterbalanced for order between participants. Air was presented not only between each of the stimulus blocks (REST), but also at the beginning and at the end of each run, as a separate condition (AIR) (see Figure 1). This design was chosen to allow the investigation of gender effects from the perspective of both donors and detectors.

#### Imaging parameters

Data were acquired using a Siemens 3T Trio whole body scanner (Siemens Medical Systems, Malvern, PA) with a circularly polarized T/R head coil. After an initial localizer scan, a high resolution (T1 weighted MPRAGE3D, resolution (RL, AP, SI) of 1.336161 mm (TI  = 1100, TR/TE  = 2100/2.74, a  = 120, FOV =  170×256×256 mm, 128×256×256 pixels, total imaging time 8:59) was acquired for anatomical registration. All fMRI data were collected as follows: single shot gradient echo EPI, TR/TE  = 2500/30 ms, 64×64 matrix, 224×224 mm FOV, 26 interleaved transverse slices (aligned to the AC-PC line) 3.5 mm thick with no gap, 1 average, flip angle = 83°.

#### Preprocessing

All image pre-processing for the analyses was implemented using the SPM5 program (Wellcome Department of Cognitive Neurology). For each participant's GE-EPI dataset: 1) Data were temporally shifted to correct for the order of slice acquisition, using the first slice acquired in the TR as the reference. 2) All GE-EPI images were realigned to each other. 3) The T1-weighted (structural) image was co-registered to the first EPI volume using a mutual information co-registration algorithm. 4) The coregistered high-resolution image was used to determine parameters (7×8×7 non-linear basis functions) for transformation into a Talairach standard space defined by the Montreal Neurologic Institute template brain supplied with SPM5. 5) This transformation was applied to the GE-EPI data, which were re-sliced to 2 mm ×2 mm ×2 mm using 7th degree polynomial approximation to sinc-interpolation. 6) The spatially normalized GE-EPI data were spatially smoothed with an isotropic Gaussian kernel (fullwidth-at-half-maximum  = 6 mm).

### Data analysis

In our previous work, the fMRI data analysis comprised the two typical levels of voxel-wise General Linear Models (a first-level participant-separable time series analysis yielding summary measures, and a second-level GLM using these measures to make statistical inference at the population level). In the first-level analysis, conditions were modeled with predictors comprising 20-second duration boxcars convolved with the default hemodynamic response function (HRF) of SPM5. The response was constructed assuming a typical 20-second HRF, since the actual time and shape of the response to alarm chomosensory stimuli is not yet known, and could not be consistently determined based on the statistical power provided by the data-set without the assumption of an underlying model. The choice was then verified to indeed outline the actual response in the amygdala, elicited by the alarm stimuli.

While this was an ideal set-up for the aim of the original study (to prove amygdala involvement in the emotional aspects of chemosensory transmission), we suspect that the assumption made in the timing and shape of the response – being hypothesis, and not data-driven – may overlook subtler aspects of the data. Indeed, our prior work showed us how significant differences in time series dynamics, which could not be pinpointed by a traditional analysis of the data (using the GLM), were better detected when performing a model-free, direct Fourier analysis of the unprocessed time series spectra [39], [40].

In the current study, while allowing our previous activation results to motivate our choice for the bilateral amygdala as our main interest areas, we henceforth embraced a model-free, direct approach to the corresponding time-series, in the sense that we made no assumptions of a canonical shape for the HRFs. This is unusual in imaging studies, because, in absence of data from a considerable amount of subjects, it is hard to make any statistical inference without an underlying model (e.g., the Baysian model used by the Statistical Parametric Mapping package). However, as explained above, using this package requires making assumptions on the shape and timing of the HRF (via the use of an appropriate choice of basis functions), based on *a priori* knowledge of this behavior. While the SPM model is safe to use for stimuli in which the response function had already been determined (such as visual, or even olfactory stimuli), in our case (of odorless chemosensory stimuli) this behavior is exactly part of what needs to be established. It is of course preferable to avoid any bias, and allow the data to drive the results. To do this, while avoiding the statistical weaknesses outlined above and any subsequent miss-interpretation, we proceeded as follows:

Directly after preprocessing, we extracted voxel-wise time series, separately for each of the four individual runs. Then, in order to increase power and reduce the noise-induced variability, we averaged the portions corresponding to blocks of stimuli of the same type. To obtain a normalization of the signal (corresponding to SPM “contrasts”), the activation was considered with respect to a “baseline,” calculated as the mean activation during the second part of the REST block before the stimulus occurred (where the impact of the preceding stimulus is least significant). The air/rest block (R) following the stimulus block was included in this average time series, for better visualization of the departure and return of the activation to this baseline. For each fixed subject, we thus obtained average voxel-wise time series for all of the stimulus types (ME, MF, FE and FF), each having 16 data-points (40 seconds).

We chose to study these time series over four regions of interest (ROIs): the bilateral amygdala (proven by our prior work to activate in both genders in response to presentation of alarm chemosensory cues) and the bilateral olfactory bulb (the primary olfactory projection area, for odors as well as for alarm chemosignals). To obtain the individual's response over the whole ROI, we averaged again, spatially, over the voxels that comprise the region. Since, in the circuitry involved in the processing of alarm chemosignals, different amygdala subnuclei are attributed different roles in existing literature, we also considered smaller sub-divisions for the amygdala: latero-basal (LB), centro-medial (CM) and superficial (SF). The anatomical masks for the amygdala and its sub-divisions were defined by using the publicly available region of interest library, the Anatomical Toolbox [45].

Figure 2 shows an example (for one person) of average activation throughout the right amygdala, along the 40 seconds of the typical MF block, followed by the respective R block. In other figures (e.g., Figure 3b), we show right amygdala activation, but this time as a *group average* evolution during and following the MF block: the male group average in blue, and the female group average in red. The error bars corresponding to each data-point refer to the distribution of the values within the respective group at that point.

We first performed a between-group Wilcoxon rank comparison, independently for each data-point – to give us a rough description of how different the evolutions of the two groups were, in response to the same type of stimuli. The rank statistics was chosen as the statistical comparison that best deals with small samples and that makes no additional assumptions on the nature or shape of the distributions. However, in order to eliminate some of the wide variability in our time series due to noise inherent to the small sample size, we built-up a slightly different measure, more innocuous to noise and which better extracts the essence of the between-groups statistics. Instead of comparing the actual time-series over each of their data-points, we averaged the time series values over small intervals, then performed the comparisons for each corresponding interval, as follows (see Figure 2): (1) first over half-blocks, obtaining 4 segments to be compared, and (2) over the two stimulus and rest blocks, rendering 2 segments and (3) over the whole two-block, activation/rest interval. (We considered a result robust when is passed all three statistical tests.) The results of these statistical comparisons are presented in Tables 1 and 2, and further explained in the Results section.
